# Bisphosphonate for spontaneous osteonecrosis of the knee: A protocol for systematic review and meta-analysis of randomized controlled trials

**DOI:** 10.1097/MD.0000000000023123

**Published:** 2020-12-04

**Authors:** Zhen Shen, Zehua Chen, Zhuoting Xie, Yanfei Xu, Tao Wang, Jiao Li, Changfei Yuan, Jinqing Liu, Xiaodong Shi, Yuanliang Ai, Wei Dong, Ying Guo

**Affiliations:** a Kunming Municipal Hospital of Traditional Chinese Medicine, Kunming, China; b The Third Affiliated Hospital of Yunnan University of Chinese Medicine, Kunming, China; c The Fifth Clinical Medical School; d The Third Clinical Medical School, Guangzhou University of Chinese Medicine, Guangzhou, China.

**Keywords:** spontaneous osteonecrosis of the knee, bisphosphonate, protocol, systematic review

## Abstract

**Background:**

Bisphosphonates are commonly used to treat spontaneous osteonecrosis of the knee (SONK), while there are no relevant systematic review or meta-analysis designed to evaluate the effects of bisphosphonates on SONK.

**Methods:**

We will identify relevant randomized controlled trials from the PubMed, EMBASE, CINAHL and China National Knowledge Infrastructure, up to March 20, 2020. Data that meets the inclusion criteria will be extracted and analyzed using RevMan V.5.3 software. Two reviewers will assess quality of the included studies by using the Cochrane Collaboration risk of bias tool. Egger test and Begg test will be used to evaluate publication bias. And Grading of Recommendations Assessment, Development and Evaluation will be employed to assess the quality of evidence.

**Results:**

In this study, we will analyze the effect of bisphosphonates on pain intensity, physical function, biochemical including alkaline phosphatase, N-terminal propeptide of type I procollagen, and C-terminal type I collagen telopeptide, radiological outcome (evaluated by using Magnetic resonance imaging) and ratio of secondary surgery for patients with SONK.

**Conclusion:**

Our findings will provide evidence for the effectiveness and potential treatment prescriptions of bisphosphonates acupuncture for patients affected by SONK.

## 1 Introduction

Osteonecrosis, as a devastating disease, can lead to end-stage arthritis of various joint including knee joint.^[1]^ Spontaneous Osteonecrosis of the Knee (SONK), first described in 1968, is a painful and relatively prevalent disease in the elderly.^[2]^ SONK manifesting as knee pain, swelling, dysfunction and even deformity,^[3]^ is a poorly understood due to unknown etiology of the condition.^[4]^ Magnetic resonance imaging (MRI) is often utilized to make a definite diagnosis at the early stage of SONK.^[5]^ With the development of the disease, it will reveal medial femoral condyle osteopenia, bone marrow edema, and consequently subchondral bone collapse when examined by using MRI. Once substantial joint surface collapse has occurred, joint arthroplasty becomes to the most appropriate treatment option.^[6,7]^ Although it is reported that unicompartmental knee arthroplasty is an excellent approach for patients with SONK,^[8]^ there are some inevitable complications, such as infection, postoperative pain, prosthesis loosening. Thus, it is critical to develop a method for preventing further progression or delaying the onset of end-stage arthritis of the knee. It is suggested that SONK is considered to be associated with subchondral insufficiency fractures, and early stage SONK is rather a result of the subchondral fracture than primary osteonecrosis.^[9]^ Promoting reconstruction and repair of fracture seems to be a potential treatment strategy for SONK.

Bisphosphonates are a group of drugs, including alendronate, ibandronate and so on, that are commonly applied in clinical practice for the treatment of osteoporosis and bone malignancies.^[10]^ Because bisphosphonates can attribute to expedite apoptosis of the osteoclast, it is beneficial to inhibit bone resorption, increase bone mineral. From this it appears that, at early stage, bisphosphonates not only can promote reconstruction and repair of subchondral fracture, but also can prevent further aggravation of the fracture, which will improve symptoms and imaging appearance for SONK.^[11]^ In a previous observation study, it is demonstrated that the incidence of secondary surgery appears to be less when bisphosphonates are given.^[12]^ However, it is reported in a randomized controlled trial (RCT) that bisphosphonate treatment has no beneficial effect compared to anti-inflammatory medication.^[13]^ Consequently, the results of bisphosphonates treatment of SONK are inconsistent, and there is insufficient evidence to support the use of bisphosphonates. The purpose of this study is to examine current evidence related to the effectiveness and safety of bisphosphonates as a treatment for SONK.

## 2 Methods

This meta-analysis will be performed according to the Preferred Reporting Items for Systematic Review and Meta-analyses.^[14]^ We have registered the protocol of this review with the Open Science Framework (OSF, https://osf.io/ychkn). The registration DOI of this study is 10.17605/OSF.IO/YCHKN.

### 2.1 Selection criteria

#### 2.1.1 Study design

In this study, all the articles of clinical RCTs evaluating the efficacy of bisphosphonates on SONK will be collected. We will include the clinical RCTs published in English or Chinese. However, the articles including full-text unavailable studies, unpublished literatures, observational studies, case series, animal experiments, qualitative studies, proceedings, conferences, comments, and reviews will be excluded.

#### 2.1.2 Patients

People who are diagnosed with an early-stage or end-stage SONK will be included in the present review. Osteonecrosis and/or bone marrow edema in the medial or lateral femoral condyles/ tibial plateau were confirmed by MRI scan. Patients suffering from secondary osteonecrosis or post-arthroscopic osteonecrosis of the knee will be excluded.

#### 2.1.3 Intervention

Five comparisons with respect to the interventions studied between experimental group and control group will be included in the present study: bisphosphonates (oral or injection) with basic treatment versus basic treatment; bisphosphonates (oral or injection) with basic treatment versus basic treatment; bisphosphonates (oral or injection) versus other treatment; bisphosphonates (oral or injection) versus no intervention.

#### 2.1.4 Outcome measures

The primary outcomes of this review will include pain intensity and physical function. Meanwhile, biochemical including alkaline phosphatase, N-terminal propeptide of type I procollagen, and C-terminal type I collagen telopeptide, radiological outcome (evaluated by using MRI) and ratio of secondary surgery will be included as secondary outcomes.

### 2.2 Search strategy

We will identify relevant studies by searching the electronic database, including PubMed, EMBASE, CINAHL and China National Knowledge Infrastructure database, up to March 20, 2020. A keyword such as “spontaneous osteonecrosis of the knee,” “SONK,” “randomized controlled trial,” “randomized, and so on. will be used to search without restrictions.

### 2.3 Study selection and data extraction

**Figure 1:**
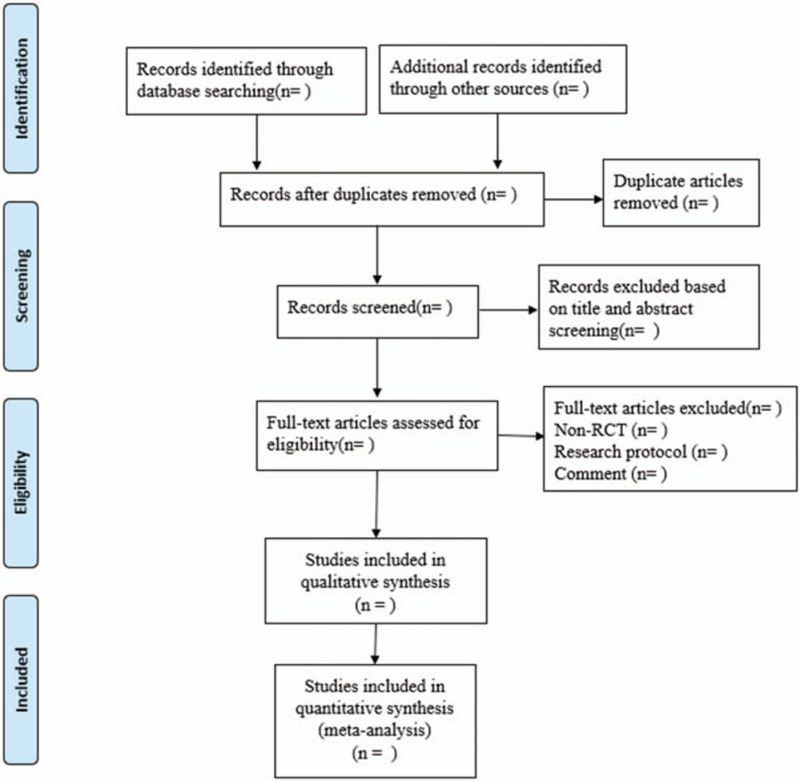
Flow chart of the search process.

All the literatures in this study will be screened by two researchers (ZH Chen and Z Shen). First, literatures will be preliminarily selected after careful reading of the topics and abstracts. Second, the uncertain documents will be screened strictly according to the inclusion and exclusion criteria after reading the full text. Subsequently, we will collect the main information of the articles, including authors’ names, publication year, age and gender of patients, study design, intervention type, intervention dose, main outcomes and sample size. During the period of screening and data extraction, if discrepancies could not be resolved through discussion, the primary reviewer would be consulted. A PRISMA flow chart will be drawn to illustrate the study selection procedure (Fig. 1).

### 2.4 Quality assessment

The quality of the included literature will be assessed by two reviewers (ZH Chen and Z Shen) table according to the risk of bias.^[15]^ The literature will be evaluated from seven aspects: sequence generation, allocation concealment, blind of participants and personnel, blind of outcome, incomplete outcome data, selective reporting and other biases. The risk of bias is divided into 3 levels: high, unclear, and low.

### 2.5 Data synthesis and analysis

We will conduct the meta system analysis of the observation indicators in the included literatures by using the review manger 5.3 software, and the results will be illustrated by the forest map intuitively. The continuous variables will be pooled by standard mean differences (SMDs) or mean differences (MDs) with 95% confidence intervals (95% CI), whereas the odds ratios (OR) will be utilized to assess the enumeration data. Heterogeneity will be assessed by the Cochran *Q*-test and *I*^2^ index.^[16]^ An *I*^2^ statistic greater than 50% is considered to be substantially heterogeneous. The fixed effects models will be employed for the meta-analysis result with low heterogeneity. However, a meta-analysis using the random effects models or a subgroup analysis will be conducted, if a substantially heterogeneous are observed. Based on the Cochrane Handbook for Systematic Reviews of Interventions,^[17]^ when the number of studies is less than 5 or studies showed substantially heterogeneous, a random-effects model should be applied. The difference is considered to be statistically significant when *P*-values is less than .05.

### 2.6 Assessment of reporting biases

Publication bias was assessed by the Begg and Egger tests.^[18]^ A *P* value<.05 in Egger test or Begg test is considered statistically significant.

### 2.7 Confidence in cumulative evidence

In addition, we will assess the quality of evidence by using grading of recommendations, assessment, development, and evaluation; version:3.6 approach.^[19]^ The quality of each evidence will be categorized into 4 levels: high, medium, low, and very low. Disagreements will be resolved by consensus.

## 3 Discussion

SONK always has a poor prognosis. Because the aetiology of SONK remains unknown, it is challenging to find the most suitable treatment for it. As is reported, SONK shows a significant decrease in regional bone density of the affected femoral condyle compared to the unaffected side.^[20]^ Morever, subchondral fracture was closely related to onset of SONK. Bisphosphonates are proved to have a positive effect on to improve bone density, and promote reconstruction and repair of fracture. However, whether it is effective and safe to utilize bisphosphonates to treat SONK is still lack of evidence. This study will be the first time to systematically review and quantify the efficacy and safety of bisphosphonate for SONK. We hope this study will provide reference for the treatment of SONK in the fields of non-operative therapies.

## Author contributions

**Conceptualization:** Zhen Shen, Zehua Chen.

**Data curation:** Yanfei Xu, Tao Wang, Jiao Li.

**Formal analysis:** Changfei Yuan, Jinqing Liu.

**Investigation:** Xiaodong Shi, Zhuoting Xie.

**Methodology:** Yuanliang Ai.

**Review sponsor:** Wei Dong, Ying Guo.

**Software:** Changfei Yuan, Jinqing Liu.

**Writing – original draft:** Zehua Chen.

**Writing – review & editing:** Zhen Shen, Miao Tian, Yuheng Li.

## Correction

Dr. Zehua Chen&s name appeared incorrectly as Zehua H. Chen and has since been corrected. The corresponding author infomration has been updated from Yuanliang Ai to Ying Guo.
